# Synchronous Gastric Gastrointestinal Stromal Tumor and Colon Adenocarcinoma: A Case Report

**DOI:** 10.1155/2014/305848

**Published:** 2014-08-17

**Authors:** Thivi Vasilakaki, Kalliroi Koulia, Aikaterini Tsavari, Elissavet Arkoumani, Efstratios Kouroumpas, Anargiros Pavlis, Georgios Christopoulos, Konstantinos Stamatiou, Kassiani Manoloudaki, Dimitrios Zisis

**Affiliations:** ^1^Department of Pathology, “Tzaneion” General Hospital of Piraeus, 1 Afendouli Avenue, 18536 Piraeus, Greece; ^2^Department of Surgery, “Tzaneion” General Hospital of Piraeus, 1 Afendouli Avenue, 18536 Piraeus, Greece; ^3^Department of Gastroenterology, “Tzaneion” General Hospital of Piraeus, 1 Afendouli Avenue, 18536 Piraeus, Greece

## Abstract

Gastrointestinal stromal tumors (GISTs) represent the majority of primary mesenchymal tumors of the gastrointestinal tract. They are generally considered to be solitary tumors and therefore the synchronous occurrence with other primary malignancies of gastrointestinal track is considered a rare event. Here we present the case of a 75-year-old man admitted to our hospital with a 10-day history of gastrointestinal bleeding. Colonoscopy revealed an ulcerative mass of 4 cm in diameter in the ascending colon. Gastroscopy revealed a bulge in the gastric body measuring 1 cm in diameter with normal overlying mucosa. Surgical intervention was suggested and ileohemicolectomy with regional lymph node resection along with gastric wedge resection was performed. Pathologic examination of the ascending colon mass showed an invasive moderately differentiated adenocarcinoma stage III B (T3N1M0). Grossly resected wedge of stomach showed a well circumscribed intramural tumor which microscopically was consistent with essentially benign gastrointestinal stromal tumor (according to Miettinen criteria). The patient did not receive additional treatment. Two years later the patient showed no evidence of recurrence or metastasis.

## 1. Introduction

Gastrointestinal stromal tumors (GISTs) are the most common primary mesenchymal neoplasms of the gastrointestinal track and they account for 2.2% of all malignant gastric tumors. GISTs can occur anywhere along the GI tract but are most common in the stomach (50–60%) and small bowel (20–25%). Colon (10%), omentum/mesentery (7%), and esophagus (5%) are less common primary sites [1–4]. Most GISTs arise from interstitial cells of Cajal. These cells are present inside and around the myenteric plexus and show both myogenic and neural differentiation, a fact that explains the immunohistochemical heterogeneity of the tumors that derive from them [1, 5, 6]. The spectrum of GIST includes neoplasms with both benign and malignant behaviour. The last has been associated with size, location, and mitotic count of the tumor. In fact, GISTs that are 2 cm or less in size can be regarded as essentially benign; however, intestinal GISTs are more aggressive than gastric GISTs of equal size, while tumors with a* KIT *exon 11 mutation are associated with a worse outcome than tumors with other KIT mutant isoforms or with no detectable mutation [7].

GISTs are generally considered solitary tumors and therefore the synchronous occurrence with other primary malignancies of gastrointestinal track is considered a rare event. Here we report a case of small gastric GIST incidentally detected during endoscopy that occurred synchronously with a primary colon adenocarcinoma.

## 2. Case Report

A 75-year-old man was admitted to our hospital with a 10-day history of gastrointestinal bleeding. He complained of a bad stomach ache for about four months; however, he reported no family history of gastrointestinal disease. Computerized tomography (CT) of the pelvis showed an obvious high-density enhancing lesion in the wall of the ascending colon. Colonoscopy revealed an ulcerative mass of 4 cm in diameter in the ascending colon. Biopsy from the mass diagnosed an invasive moderately differentiated adenocarcinoma. Gastroscopy revealed a bulge in the gastric body measuring 1 cm in diameter with normal overlying mucosa. Mucosal biopsies diagnosed chronic gastritis; however no evidence of* Helicobacter pylori* infection was found. Endoscopic ultrasound of the gastric bulge showed a 1 × 1 cm lesion involving the gastric wall.

**Figure 1:**
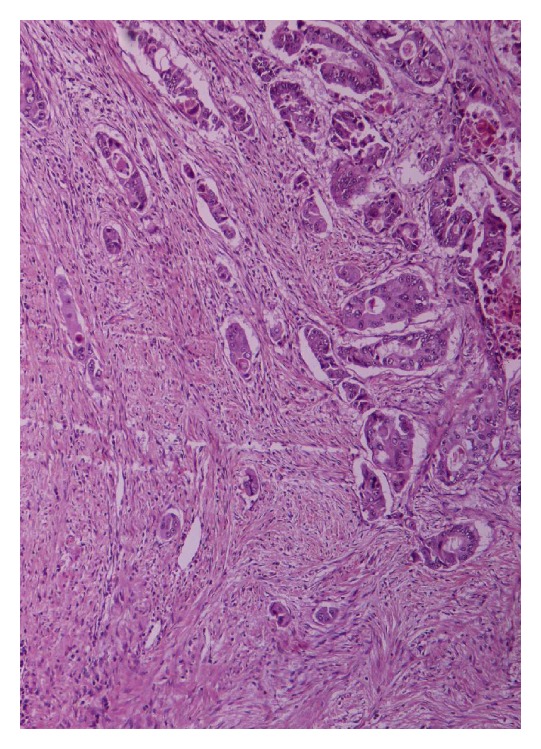
Colon adenocarcinoma (H/E ×100).

Surgical intervention was suggested and ileohemicolectomy with regional lymph node resection along with gastric wedge resection was finally performed. Pathologic examination of the ascending colon specimen revealed a stage III B disease (T3N1M0 according to TNM classification) (Figure 1). Only one out of the six resected lymph nodes showed tumor metastasis.

**Figure 2:**
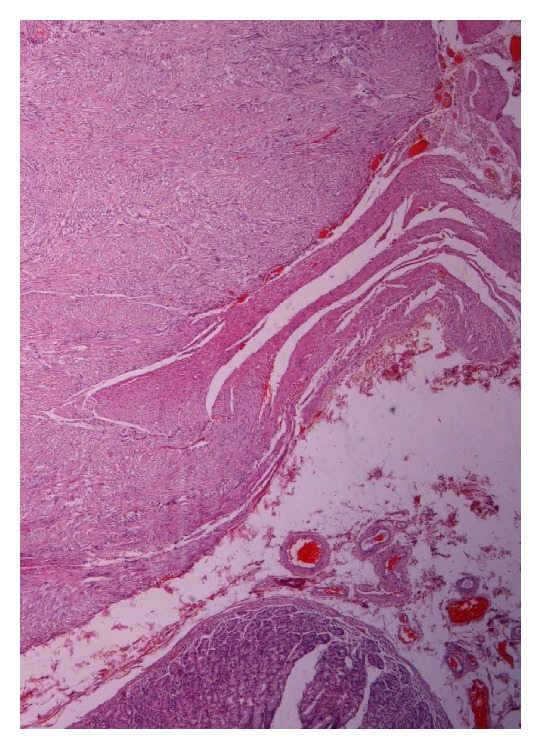
Gastric GIST (H/E ×40).

**Figure 3:**
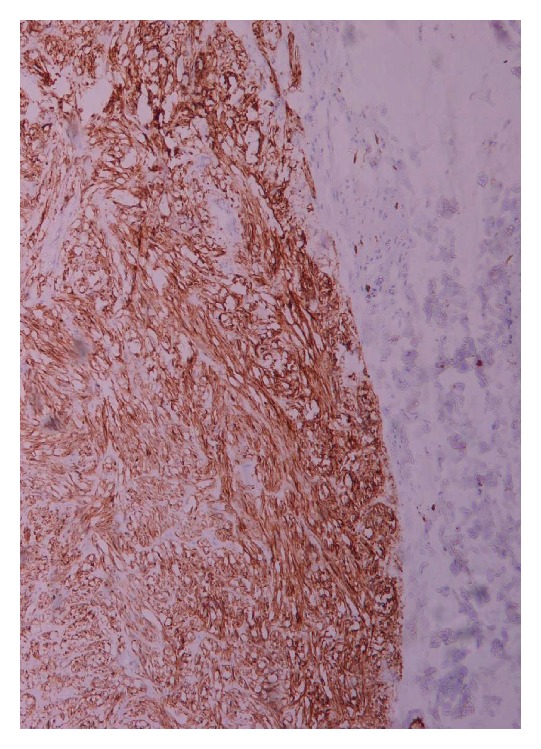
Gastric GIST, CD117 positive (×100).

Grossly resected wedge of stomach showed a well circumscribed intramural tumor which microscopically was consistent with an essentially benign gastrointestinal stromal tumor (according to Miettinen criteria). The lesion involved the muscularis propria with variable submucosal extension (Figure 2). The immunohistochemical study showed that the tumor's cells were positive for CD117 and CD34 and negative for actin, desmin, and S100p (Figure 3). The Ki67 labelling index was very low. The mitotic activity was <1 mitosis/50 high power field (HPF). The patient did not receive additional treatment and he was monitored with annual abdominal CT. Two years later the patient showed no evidence of recurrence or metastasis.

## 3. Discussion

Gastrointestinal cancer is common and is a significant cause of morbidity and mortality. The synchronous occurrence of two malignancies is not uncommon, but such occurrences often pose diagnostic and therapeutic challenges. Moreover, the coexistence of malignancies of different histological origin along the GI track, raises questions regarding the natural history and the pathophysiology of GI cancer.

The majority of GISTs are sporadic and tumor multiplicity is considered an exceptional finding limited to specific conditions such as hereditary GIST, paraganglioma, and Carney's triad syndromes [8]. Beyond these entities, the occurrence of multiple distinct tumors is conventionally interpreted as indicative of metastatic spread from a primary lesion [8]. However, neoplasms with histology and immunohistochemistry similar to GISTs may occur outside the gastrointestinal track in the abdomen or in the retroperitoneum. These tumors must be defined as extragastrointestinal stromal tumors since they display no connection with the gastric of intestinal wall [5, 9]. Actually, GISTs have a broad morphological spectrum and show positivity for CD117 which appear as cytoplasmic, as membrane-associated, or sometimes as perinuclear dots. However, a small minority (<5%) especially GISTs with mutant PDGFRA may have very limited, if any, positivity [5]. On the other hand, GISTs have been associated with synchronous primary neoplasms of different histogenesis. The most common are carcinomas of gastrointestinal track. Other coexistent tumors include carcinomas of breast, lung, kidney, prostate, female genital track, soft tissue sarcomas, lymphoma, and malignant melanoma [1, 3]. However, the etiology of synchronous occurrence of GISTs with histologically unrelated tumors still remains unclear [1].

Activation of the KIT receptor tyrosine kinase is integral to the development of many GISTs. This activation involves a mutation within the c-*kit *gene. Most GISTs (approximately 70%) harbour a mutation in exon 11. In about 15% of cases there is a mutation in exon 9 of* KIT*, while, less commonly (<10%), mutations occur in exons 13 and 17 [10]. These mutations (including deletions and point mutations) result in gain of function. Thus, KIT signalling is constitutively activated resulting in downstream phosphorylation in the signal transduction pathway, ultimately leading to increased cellular proliferation [10]. However, in several malignancies—including colorectal adenocarcinomas—alterations of the tyrosine kinase activity is associated with advanced disease. In fact, deregulation of the TK receptor enhances the ability of cancer cells to migrate, initiating thus the metastatic cascade [11]. While it is clear that activating mutations in KIT are an early event in GISTs, it is not known whether they can serve as an initiating oncogenic event in colorectal cancer [10, 11]. The fact that in this particular case a small GIST (with very low malignant potential) coexisted with a large primary colon adenocarcinoma suggests an association between adenocarcinoma and GIST; however this association does not seem to have an impact on survival. Given the limited number of cases further studies are required to clarify the molecular and genetic mechanisms of carcinogenesis.

In conclusion, the patients with synchronous GIST and other neoplasms present diagnostic difficulties because it is not always possible to recognize a coexisting tumor preoperatively.
